# Reflections on the Field of Human Genetics: A Call for Increased Disease Genetics Theory

**DOI:** 10.3389/fgene.2016.00106

**Published:** 2016-06-08

**Authors:** Steven J. Schrodi

**Affiliations:** ^1^Marshfield Clinic Research Foundation, Center for Human GeneticsMarshfield, WI, USA; ^2^Computation and Informatics in Biology and Medicine, University of Wisconsin-MadisonMadison, WI, USA

**Keywords:** disease genetics, theoretical model, human genetics, GWAS (genome-wide association study), complex diseases, statistical genetics and genomics

## Abstract

Development of human genetics theoretical models and the integration of those models with experiment and statistical evaluation are critical for scientific progress. This perspective argues that increased effort in disease genetics theory, complementing experimental, and statistical efforts, will escalate the unraveling of molecular etiologies of complex diseases. In particular, the development of new, realistic disease genetics models will help elucidate complex disease pathogenesis, and the predicted patterns in genetic data made by these models will enable the concurrent, more comprehensive statistical testing of multiple aspects of disease genetics predictions, thereby better identifying disease loci. By theoretical human genetics, I intend to encompass all investigations devoted to modeling the heritable architecture underlying disease traits and studies of the resulting principles and dynamics of such models. Hence, the scope of theoretical disease genetics work includes construction and analysis of models describing how disease-predisposing alleles (1) arise, (2) are transmitted across families and populations, and (3) interact with other risk and protective alleles across both the genome and environmental factors to produce disease states. Theoretical work improves insight into viable genetic models of diseases consistent with empirical results from linkage, transmission, and association studies as well as population genetics. Furthermore, understanding the patterns of genetic data expected under realistic disease models will enable more powerful approaches to discover disease-predisposing alleles and additional heritable factors important in common diseases. In spite of the pivotal role of disease genetics theory, such investigation is not particularly vibrant.

## Introduction

Development of human genetics theoretical models and the integration of those models with experiment and statistical evaluation are critical for scientific progress. This perspective argues that increased effort in disease genetics theory, complementing experimental, and statistical efforts, will escalate the unraveling of molecular etiologies of complex diseases. In particular, the development of new, realistic disease genetics models will help elucidate complex disease pathogenesis, and the predicted patterns in genetic data made by these models will enable the concurrent, more comprehensive statistical testing of multiple aspects of disease genetics predictions, thereby better identifying disease loci. By theoretical human genetics, I intend to encompass all investigations devoted to modeling the heritable architecture underlying disease traits and studies of the resulting principles and dynamics of such models. Hence, the scope of theoretical disease genetics work includes construction and analysis of models describing how disease-predisposing alleles (1) arise, (2) are transmitted across families and populations, and (3) interact with other risk and protective alleles across both the genome and environmental factors to produce disease states. Theoretical work improves insight into viable genetic models of diseases consistent with empirical results from linkage, transmission, and association studies as well as population genetics. Furthermore, understanding the patterns of genetic data expected under realistic disease models will enable more powerful approaches to discover disease-predisposing alleles and additional heritable factors important in common diseases. In spite of the pivotal role of disease genetics theory, such investigation is not particularly vibrant. Currently, activities in human disease genetics are primarily centered upon large-scale empirical studies and, to a lesser extent, statistical methods, with limited contribution to theory.

## Background and framework

Broadly speaking, scientific progress is predicated on a robust interplay between three activities: (1) empirical experimentation and observation, (2) the development of theoretical models and extraction of predicted patterns thereof, and (3) the statistical evaluation of the probabilistic correspondence between the predicted patterns and empirical data. Highly impactful discoveries can certainly occur in the absence of formalization of these activities, but these three aspects are nonetheless critical. To exemplify, consider the relatively recent remarkable finding of complex, low-level admixture between modern humans and archaic humans (Green et al., 2010; Reich et al., 2010; Gronau et al., 2011; Li and Durbin, 2011; Sankararaman et al., 2016). This discovery was made through heroic efforts to isolate, sequence, assemble, and align archaic DNA from Neanderthal and Denisovan remains. In parallel, predictions of genetic architecture, divergence patterns, and shared chromosomal regions from admixture models were developed using both molecular phylogenetics and population genetics theory involving mutation, genetic drift, migration, and demographics. Lastly, correspondence between the observed genetic data and theoretical predictions were accomplished through a variety of likelihood-based, Bayesian, and Fisherian approaches. It is not overreaching to claim that this advance of our scientific knowledge hinged on careful empirical observations/experiments, the development of population genetics theory, and the formal evaluation of rich theoretical predictions against observed data through rigorous statistical methods. The most casual of observers will note legions of additional examples of this paradigm from a diverse set of scientific fields such as particle physics (Glashow, 1961; Higgs, 1964; Weinberg, 1967), mechanics (Einstein, 1916; Schrodinger, 1926), enzyme kinetics (Michaelis and Menten, 1913), semiconductors (Hall, 1879; Wilson, 1931; Mott, 1938; Schottky, 1938), atomic chemistry (Hund, 1926; Mulliken, 1932; Huckel, 1934), classical genetics (Mendel, 1866; Fisher, 1918), heredity and evolution (Fisher, 1930; Wright, 1932; Price, 1970), population genetics (Hardy, 1908; Weinberg, 1908; Hudson, 1982, 1983; Kingman, 1982a; Gillespie, 1993, 2000), and predator-prey ecology (Lotka, 1925). Across these and many other fields, theoretical work is a dynamic component of the scientific process: unexplained empirical phenomena motivate new theory, often in a relatively seamless manner, and, conversely, predicted patterns stemming from mechanistic models are digested by experimenters and promptly tested, leading to an expeditious and efficient expansion in our understanding of these phenomena.

Comparatively, disease gene mapping has grown a relatively barren landscape of theory. Largely motivated by the desire to impact clinical practice, human disease genetics has historically been a highly pragmatic field where technological advancement in genotyping and sequencing has spurred large-scale studies and the bulk of quantitative work has focused on statistical methods of analysis, rather than a more equitable partition of statistics and theory. This has continued despite instances where profound shifts in approaches have been driven by insights from theory. For example, an extended stagnation in mapping common, complex diseases was fractured by theoretical developments in the mid- to late-1990s showing that high density genotyping using population-based samples would have dramatically increased power to detect high frequency disease-predisposing alleles of moderate effect sizes, motivating the GWAS paradigm from an overly-simplistic disease genetics model (Kaplan et al., 1995; Risch and Merikangas, 1996; Long et al., 1997; Xiong and Guo, 1998; Kruglyak, 1999; Long and Langley, 1999).

Although not commonplace, there are other historical examples of disease genetics theory driving accelerated progress in human genetics, including the heterozygote selective advantage theory of malaria and sickle-cell disease (Allison, 1954), that complex diseases do have a heritable component (Steinberg et al., 1951; Pickering, 1978; Debray et al., 1979; Kendler and Diehl, 1993; DeBraekeleer, 1991; Lynn et al., 1995; Stein et al., 2005), that large multiplex families were ideal for linkage studies of diseases under Mendelian disease models (Thompson, 1978; Botstein et al., 1980), and the more diffuse impact of theoretical ideas from population genetics such as allele frequency spectra being highly skewed toward very rare alleles (Ewens, 1972; Watterson, 1975; Slatkin and Rannala, 1997; Long and Langley, 1999; Eyre-Walker, 2010; Hudson, 2015)—accentuated in rapidly expanding populations, haplotypes exhibiting block-like structure in LD patterns (Hill and Robertson, 1968; Hudson and Kaplan, 1985; Nothnagel et al., 2002; Wiuf and Posada, 2003), population bottlenecks followed by expansion accentuates allelic dominance effects on fitness (Balick et al., 2015), and alleles with deleterious effects on fitness originating more recently than those neutral with respect to fitness given the same allele frequency (Maruyama, 1974; Ziezun et al., 2013). Theoretical work has been done on applying the highly polygenic, additive model to common diseases, offering some testable predictions (Yang et al., 2010, 2011a; Vinkhuyzen et al., 2013; Loh et al., 2015). Additional, useful efforts have focused on widespread epistatic interactions (Hodge, 1981; Neuman and Rice, 1992; Majewski et al., 2001; Zuk et al., 2012). However, competing theoretical models of common disease genetics are sparse even though both history and reason argue for a more vigorous theoretician community and heightened interaction between theory, experiment and statistical methods.

## What constitutes a useful theory of disease genetics?

Although many areas of investigation rightly fall under this rubric, the general focus should be the development of testable models that describe the set of heritable factors and interactions that generate disease states. This includes allele and genotype frequencies, numbers of susceptibility loci, numbers and types of susceptibility alleles, penetrances, epistatic interactions, properties of familial transmission, and effect modification with environmental variables. It is important to draw a distinction between the genetics that predispose an individual to a disease and the genetic repertoire that underlies the predisposition to a disease across a population of affected individuals, for we do not know the extent in which each individual's disease etiology is unique for complex disease phenotypes. That is, although it is well established from population-level analyses that complex diseases are polygenic, we currently have little evidence that definitively speaks to the level of allelic and locus heterogeneity in any complex disease. What set of genotypes at a set of loci are sufficient to generate disease in an individual? What is the variation in these disease-predisposing sets of genotypes and loci across diseased individuals? Do the alleles co-segregate with disease states across relatedness structures? Coherent, useful theories of disease genetics must address these questions.

Theoretical population genetics, with numerous practitioners using coalescent theory and similar tools to study the maintenance of alleles in populations and elucidate the evolutionary forces responsible for genetic variation, is relatively advanced (Kaplan et al., 1988; Hudson, 1991; Charlesworth et al., 1997; Calafell et al., 2001). As population genetics is concerned with the dynamics and distributions of alleles in populations (Ewens, 1972; Moran, 1975; Watterson, 1975; Kingman, 1982a,b; Charlesworth and Jain, 2014; Greenbaum, 2015), the relevance of population genetics theory to disease gene mapping—particularly for case-control association studies, fine-scale mapping, and population stratification—is undeniably clear, with several important advances demonstrating applicability (Pritchard et al., 2000; Morris et al., 2002; Molitor et al., 2003; Burkett et al., 2014). However, population genetics theory, in and of itself, is inadequate to serve as a complete theory for modeling the disease genetics: (1) coalescent theory is largely concerned with samples of random chromosomes from a population, rather than from disease-affected individuals; (2) there is limited focus on the treatment of related individuals; (3) whereas population genetics aims to delineate the relative impact of natural selection, genetic drift, mutation and demographic effects, diseases have a complex, enigmatic relationship to fitness—some diseases may result from mutation-selection balance, other diseases may carry susceptibility genes that are neutral with respect to selection, while some disease genes may be subjected to directional selection, and many diseases, such as type 2 diabetes (Hu, 2011), may result from a shift to a modern environment; (4) theoretical population genetics concentrates on the dynamics of individual loci in isolation; and (5) somatic mutations and heritable epigenetic factors, which play important roles in at least some common diseases, are often not the subject of mainstream population genetics theory.

Similarly, the theoretical models from quantitative genetics are also problematic in their direct applicability to investigations of disease genetics architecture. These models are almost exclusively direct derivatives of the infinitely polygenic, miniscule additive effects model (IPMAE model) (Falconer and MacKay, 1996; Frank, 2011). Often, when applied to a dichotomous outcome, a threshold (Wright, 1934) or liability function (Falconer, 1965; Curnow and Smith, 1972) is overlaid on the IPMAE model. Historically, work on the IPMAE model was designed for the study of quantitative traits, such as livestock lean body weight and crop yield, in agriculturally important organisms and specifically-designed pedigrees to assess measures such as breeding values (Falconer and MacKay, 1996; Lynch and Walsh, 1998). Although this model carries utility for analysis of quantitative traits in general populations, and the application to human disease is strongly argued by some (Hill et al., 2008; Plomin et al., 2009), whether or not the coupling of a liability function with the IPMAE model is indeed the appropriate model of allelic architecture for any dichotomous complex disease is currently unknown. Many, if not most disease physiologies are fundamentally different than naturally-occurring phenotype variation investigated by quantitative geneticists, and it is reasonable to assume that their underlying allelic architecture also differs. Recently, several have strongly argued against the continued use of the IPMAE model for the purpose of dissecting complex diseases (Nelson et al., 2013; Génin and Clerget-Darpoux, 2015). Although I personally favor models other than the IPMAE model for complex diseases, I do not think that either theoretical nor empirical evidence is currently sufficient to completely dismiss the IPMAE model. It is certainly possible, *a priori*, that tens or hundreds of thousands loci across the genome harbor alleles of very small effect sizes, all marginally contributing to additively increase disease risk. Moreover, many types of models may appear to have additive and nearly independent effects as those effect sizes become small. If complex diseases are a conglomeration of distinct physiological entities with their own genetic etiologies, erroneously aggregated by physicians, it appears possible that the IPMAE model may be reasonable, at least for interpreting data from population-based studies. If molecular networks are highly redundant and numerous pathogenic changes are necessary to compromise the function of these networks, then the IPMAE model might be appropriate. So, rather than disbanding the IPMAE model entirely, a prudent direction would be encouraging the development of alternative theoretical models. A competitive marketplace of disease genetics models is a critically important cog in the unraveling the genetic architecture of all diseases. Certainly, the correspondence between IPMAE predictions and experimental data will be the ultimate arbitrator. Empirically, the jury is mixed with some studies offering moderate evidence of consistency between genetic association data and the IPMAE model (Yang et al., 2010, 2011a; Vinkhuyzen et al., 2013; Bulik-Sullivan et al., 2015; Loh et al., 2015), while others do not (Ritchie et al., 2001; Kirino et al., 2013; Ridge et al., 2013; Fritsche et al., 2014), and familial data has yet to definitively support or refute the model. Of note, a useful global measure of the magnitude of polygenic inheritance has been discussed by Yang et al. (2011b). Interestingly, testing polygenetic architecture models on GWAS data for four complex diseases—rheumatoid arthritis, celiac disease, myocardial infarction/coronary artery disease, and type 2 diabetes—using Bayesian Approximate Computation, Stahl and colleagues estimated the joint density of the number of independent disease-predisposing SNPs and the liability-scale variance explained showing consistency with models using roughly 2000 SNPs (Stahl et al., 2012).

Within human genetics, the majority of models of common disease genetic architecture used in practice fall into two overly-simplistic camps: (1) monogenic and two-locus models with a biallelic markers and typically one of four classical modes of inheritance (fully dominant, fully recessive, additive, or multiplicative), and (2) direct derivatives of the IPMAE model. One only has to go as far as to look at commonly-used power calculators for genetic association or linkage studies to observe this rather ubiquitous, long-standing, yet fairly impotent state of affairs. Parametric linkage studies using monogenic models produced spurious results for complex diseases (Génin and Clerget-Darpoux, 2015). Much of their use has resulted from convenience—both the monogenic/two-locus models and the IPMAE model are mathematically tractable and other, more realistic models may necessitate complex mathematical treatment or computational approaches. Not only do these two classes of models represent the ends of a wide spectrum of models, but this limited number of disease genetics models is symptomatic of an anemic theoretical effort. That said, what we have learned about the properties and dynamics of the IPMAE (Blangero et al., 2013; Zhou et al., 2013) and monogenic/two-locus models (Li and Reich, 2000; Zaykin et al., 2006; Schrodi et al., 2007; Zaykin and Shibata, 2008) will serve us well for the development of the next generation of theoretical disease genetics models. For example, the finite, additive polygenic model relaxes from the extremely large number of disease loci assumption of the IPMAE (Cannings et al., 1978; Lange, 1997). Further, new statistical approaches, explicitly harnessing theoretical models of polygenic inheritance to better understand genetic variation of complex traits are starting to be developed (Zhou et al., 2013). In my view, finite rare allele models of moderately high effect sizes, high allelic, and high locus heterogeneity with effect modification by genetic background deserve attention. Importantly, very recent results from simulations appear to favor incomplete recessivity models for complex trait etiologies, demonstrating consistency with both realistic population genetic models, heritability data, and GWAS findings (Sanjak et al., 2016). Such work suggests prioritizing tests of recessive modes of inheritance and compound heterozygosity testing for common disease mapping.

While it is undeniable that substantial biological insights and clinical utility have resulted from identifying alleles truly associated/linked with complex diseases (Sabbagh and Darlu, 2006; Roychowdhury and Chinnaiyan, 2013; Bottini and Peterson, 2014; Kavanaugh et al., 2014; Everett et al., 2015; Lueck et al., 2015), we are currently in the infancy of understanding disease genetics where prediction of any common, complex disease is not yet clinically practicable (Schrodi et al., 2014), and efficacious, highly targeted therapies are sparse. One bright point for disease prediction, borrowed from quantitative genetics and work on highly polygenic additive models, is the use of best linear unbiased prediction (BLUP) (Speed and Balding, 2014; Vilhjalmsson et al., 2015). That said, the overall lack of realistic theoretical models dramatically hinders our progress, for powerful experimental designs and analysis techniques could be optimized to suit the predictions of such models. Yet the tools are available to make significant theoretical inroads. Data processing approaches (Fan et al., 2014) and machine learning has become incorporated into development of genetic models and their evaluation (Libbrecht and Noble, 2015). Graphical modeling programming software are well-developed (Hall et al., 2009). In addition, the investigation and use of causal models may also advance human genetics theory (Pearl, 2000; Madsen et al., 2011a,b). Fast Markov-chain-Monte-Carlo algorithms to screen complicated, vast parameter spaces are accessible. And, most importantly, the accumulated results from multiplex linkage studies, affected sibling pair studies, studies assessing disease concordance between relative pairs of varying relatedness, twin studies, family-based transmission/disequilibrium studies, GWAS, and familial and population-based sequencing studies are available. Moreover, high-throughput genotyping and sequencing have painted a detailed picture of the raw materials from which disease genetics are sampled: the allele frequency spectrum and LD patterns. Disease genetics models must be consistent with these results. Ideally, an abundant assortment of viable theoretical disease genetics models will be developed, generating informative, distinguishing predictions. These predictions can then be tested against the accumulated patterns of genetic data, producing posterior probabilities, or likelihoods for each model. As the empirical data accumulates, the posterior probability density across the parameter space of the models will indicate those models with reasonably high posterior probabilities, with many models being ruled out. Not only would such work illuminate plausible etiological models of complex diseases, but it would suggest highly-powered experimental designs and statistical methods. In particular, with the determination of likely disease genetics models, one could harness a variety of predicted patterns to improve the discovery and assessment of casual loci.

A more detailed example may provide additional weight and clarity to this argument. Consider a standard common disease case/control GWAS study. With notable exceptions of issues such as clustering of subjects using dimensional reduction methods (Price et al., 2006), most such studies are designed and analyzed to solely test the simple hypothesis of independence between disease status and genotype frequencies at single sites. However, formal disease genetics models could offer a wealth of predictions concerning genetic architecture patterns in the data: (1) Diseases with early onset and probable ancestral effects on fitness predict selection against disease-predisposing alleles which would generate departures from neutrality as measured by metrics such as Tajima's (1989). (2) Departures from Hardy-Weinberg Equilibrium differ between cases and controls under several disease models (Nielsen et al., 1999). (3) The linkage disequilibrium patterns within cases at the susceptibility locus are expected to differ from those patterns observed in controls (Zaykin et al., 2006; Schrodi et al., 2007; Pan, 2010). (4) The decay of disease association with declining linkage disequilibrium between a causal site and closely-linked markers follows a particular form (Lai et al., 1994; Pritchard and Przeworski, 2001; Garcia et al., 2008; Schrodi et al., 2009; Maadooliat et al., 2016). (5) Cases are expected to exhibit increased sharing of chromosomal segments compared to controls (Houwen et al., 1994; Te Meerman et al., 1995; Browning and Thompson, 2012). (6) Models generating allelic heterogeneity such as the rare allele/large effect (RALE) model suggest investigating multiple predisposing sequence variants segregating at each gene/functional motif (Personal communication with Ray White, 2000-2010; Terwilliger and Göring, 2000; Pritchard, 2001; Thornton et al., 2013) and perhaps testing for linkage. Little imagination is necessary to presume that additional, highly useful predicted genetic patterns exist under disease genetics models, hereto underutilized. So long as the disease genetics model is sufficiently accurate or the predictions are robust across models, concurrently testing the rich panoply of theoretical predictions extracts increased information, enabling more refined, credible, and localized discovery of pathogenic alleles. Notably, Agarwala and colleagues have conducted excellent work in this area for type 2 diabetes (Agarwala et al., 2013). They have used a combination of simulation results and results from affected sibling linkage studies, GWAS, a polygenic score logistic regression, and sequencing studies to reduce the model space of possible architecture models. They foresee further reduction in the space of possible models being dependent on the findings from very large-scale sequencing studies. I applaud this considerable effort and hope that further work in this area is strongly supported.

There are similar implications for such theoretical disease genetics when applied to family-based studies. Predictions from realistic disease genetics models enable the coherent exploration of critical questions such as (1) Is the distribution of chromosomal regions shared by affected individuals indicative of disease loci? (2) Are the observed phenotypic variance within families and familial aggregation patterns consistent with specific disease genetics models? (3) Are the transmission patterns within families consistent with disease genetics models? And (4) Given a specific disease genetics model, what is the optimal size of family structure for finding chromosomal regions linked and associated with complex diseases (i.e., siblings, multiplex families, founder populations, or general populations)? Just as with population-based studies, the development of theoretical models of disease genetics illuminates the path to jointly testing numerous observed genetic patterns within familial structures. Wray and Goddard have started to explore some of these issues and have shown that three disease models are roughly consistent with data on disease risk in relatives (Wray and Goddard, 2010).

## Related areas of theory development

One area of active research that would profit from the advancement of disease genetics theory is the development of fine mapping methods to identify causal variants within a disease-associated region. Discovering causal variants is critically important for several reasons, most notably that expensive, time-consuming, follow-up laboratory experiments are predicated on which gene or functional motif are indicted by the genetic evidence. This problem of identifying disease-causing variants in regions of often complex linkage disequilibrium patterns and allelic heterogeneity, although dramatically understudied in the past, has now been increasingly recognized as being vital in the human genetics toolbox. One example is the fine-mapping method of Maller and colleagues which uses a ranked set of Bayes factors (one for each polymorphism in an associated region) (Wellcome Trust Case Control Consortium et al., 2012). Other approaches include Bim-Bam (Servin and Stephens, 2007), CAVIAR (Hormozdiari et al., 2014), CAVIARBF (Chen et al., 2015), coalescent-based methods (Graham, 1998; Morris et al., 2002; Zollner and Pritchard, 2005), and PAINTOR (Kichaev et al., 2014), which incorporates functional information probabilistically. While I applaud these excellent, thoughtful methods, as a generalization these approaches are statistically appropriate and easily interpretable, but use overly simplistic disease genetics models. If we held a more complete understanding of the theoretical properties of alleles that underlie complex diseases and their correlation patterns with linked variants, one could incorporate this information into more powerful fine mapping methods.

Aside from the need for the development and analysis of DNA-based models of disease predisposition, similar models of other heritable factors involved in pathogenesis such as inherited RNA pools, histone acetylation, and DNA methylation effects are essential for the rapid advancement of disease genetics. It is becoming increasingly clear that epigenetic factors play a role in heritable diseases (Uddin et al., 2010; Williams et al., 2010; Allum et al., 2015; Montano et al., 2016). However, just as clear is the near absence of theoretical models describing epigenetics as an etiological factor in diseases. Several simple questions need investigation: What are the probabilistic laws that govern the transmission of these epigenetic factors? That is, what is the distribution of probabilities that a given epigenetic state is transmitted to a subsequent generation? How do these probabilities attenuate across multiple generations? What are the frequencies of various epigenetic changes and the corresponding effects on disease risk? What is the fraction of an individual's disease risk that is generated by epigenetic changes? And how is this fraction distributed across a population? Development of these theoretical models will allow for the calculation of testable predictions and aid in the construction of more powerful experimental designs.

## Conclusion

To be balanced, there certainly are efforts in human genetics theory, several of which have been discussed, enabling statistical methods that harness informative theoretical predictions (Reich and Lander, 2001; Zhu et al., 2015). The crux of the argument made here, however, is one of degree: Additional training of human geneticists in theoretical models, complementing, and motivating statistical methods, would be beneficial. Additional construction of disease genetics models to evaluate against empirical data is vitally needed. Additional work on identifying the patterns of genetic data expected under theoretical models is essential. Additional evaluation of empirical data from large multiplex families, affected sibling pairs, isolated populations, founder populations, transmission-based tests, GWAS, whole genome/exome sequencing studies in families, and population-based sequencing studies to determine which disease models are supported and which can be excluded based on these results would be highly productive. And additional interplay between theory, experiment, and analysis is critical. My central thesis is that funding and effort must be balanced in a way that produces complementarily functioning triad of theory, experiment, and statistics, so that the entire field of human genetics moves forward unabated. Currently, experimental studies and statistical methods are clearly active and highly-functioning subfields, whereas, the field has a dearth of theoretical disease genetics models, impeding the entire disease gene mapping enterprise. The timing is ideal for the institution of these changes. We have amassed vast amounts of genetic data for many hundreds of common diseases and rarer, related conditions and yet the heritable causes of each common disease remain poorly understood. Colossal, expensive shifts in focus have been historically driven by simplistic, undeveloped theoretical models, e.g., common disease/common variant hypothesis (Reich and Lander, 2001), so it may be more fruitful for our field to further develop resources and capabilities that generate more fully developed theoretical models of disease genetics. Perhaps it is time for a new field of theoretical disease genetics.

## Author contributions

The author confirms being the sole contributor of this work and approved it for publication.

## Funding

This work was supported by generous donors to the Marshfield Clinic Research Foundation, a pilot grant award from the NIH-NCATS/University of Wisconsin-Madison Institute for Clinical and Translational Research (UL1TR000427) and NIMH RO1MH097464. The content is solely the responsibility of the author and does not necessarily represent the official views of the National Institutes of Health.

### Conflict of interest statement

The author declares that the research was conducted in the absence of any commercial or financial relationships that could be construed as a potential conflict of interest.
